# A systematic review and modeling of the effect of bacteriophages on *E. coli* O157:H7 reduction in vegetables

**DOI:** 10.1016/j.heliyon.2023.e22961

**Published:** 2023-11-28

**Authors:** Maryam Safarirad, Mohsen Shahdadi, Enayat Berizi, Seyed Mohammad Mazloomi, Saeid Hosseinzadeh, Maryam Montaseri, Zahra Derakhshan

**Affiliations:** a Student Research Committee, Department of Food Hygiene and Quality Control, School of Nutrition and Food Sciences, Shiraz University of Medical Sciences, Shiraz, Iran; b Nutrition Research Center, Department of Food Hygiene and Quality Control, School of Nutrition and Food Sciences, Shiraz University of Medical Sciences, Shiraz, Iran; c Department of Food Hygiene and Public Health, School of Veterinary Medicine, Shiraz University, Shiraz, Iran; d Research Center for Health Sciences, Institute of Health, Shiraz University of Medical Sciences, Shiraz, Iran; e Department of Environmental Health Engineering, School of Health, Shiraz University of Medical Sciences, Shiraz, Iran

**Keywords:** *E. coli* O157:H7, Bacteriophages, Vegetables, Systematic review, Multivariate regression

## Abstract

Prevention and control of food pathogens are important for public health and *E. coli* O157:H7 infections are known as one of the most important food-borne bacterial diseases transmitted to humans. Vegetables can be a major source of *E. coli* O157:H7 bacteria. Bacteriophages have been considered in recent years as a natural method for controlling pathogens with minimal damage to the quality of vegetables. The performance of these natural antimicrobial agents is affected by various factors including time, temperature, phage and bacterial dose, method of phage application and origin of phages. The aim of the present study was to conduct a systematic review of the works that have examined the effect of different factors to reduce *E. coli* O157:H7 bacteria by its specific phages and model their effect. In our study, 10 articles were chosen after applying the inclusion and exclusion criteria mentioned in the methodology. The multivariate regression results showed that time, temperature, and method of phage application revealed a positive influence on the phage function, and with each unit of increase, the *E. coli* O157:H7 reduction increases by 0.4 %, 3 % and 0.94 % respectively, and 6 % for phage dose, but not statistically significant (P = 0.44). In addition, commercial-type phages were more effective than wild-type phages and this result was statistically significant (Beta = 0.99; P = 0.001). The results of this study indicate that the various factors, such as temperature, time, method of phage application and type of vegetables can play an important role to reduce *E. coli* O157:H7 in vegetables.

## 1 Introduction

Today, in order to maintain a healthy diet, eating fruits and vegetables is essential because they are rich in nutrients. However, these products contain a wide range of microorganisms that can be divided into three categories: beneficial, neutral and pathogenic [1,2]. Outbreaks of food-borne diseases, especially from consumption of raw and fresh vegetables, are a major worldwide concern [3]. In 2011, 33 % of food-borne diseases reported by the Center for Disease Control and Prevention (CDC) were related to these products [4]. In recent years, there have been numerous outbreaks of plant-related foods around the world. Therefore, in an effort to ensure food safety, a variety of strategies to reduce the risk of food contamination have been explored [5,6].

*Escherichia coli* O157:H7 is known to be a major foodborne pathogen that can cause gastroenteritis in humans [7]. According to the US Food and Drug Administration (USDA), this important food pathogen is responsible for about 62,000 illnesses a year in the United States alone, with an estimated annual cost of $ 0.7 billion. Infections caused by this bacterium often lead to dysentery and can lead to kidney failure called hemolytic-uremic syndrome (HUS) in ∼5.0 % of patients [7,8].

Investigation and control of the toxins from this bacterium in food products is currently a major challenge in the food industry, which uses various methods to control contamination. Irradiation, chemical disinfectants and pasteurization are some of the common methods in the industry to control this food pathogen. However, these methods may also kill beneficial bacteria contained in the food. Several studies have shown that conventional disinfection processes have significant disadvantages for reducing the pathogen load on vegetables [9]. Additionally, these treatments often effect the organoleptic properties of the food and may have destructive environmental effects [8]. Hence, new types of efficacious treatments, without these secondary undesirable effects need be developed.

Bacteriophages, hereafter referred to as phage are probably the most abundant microorganisms on earth [10,11]. The use of phage to reduce or eliminate pathogens, a from of biological control, has recently received much attention [12]. In fact, several studies have focused on phages as a promising alternative to eradicating pathogens. Various factors such as temperature, time, the method of phage application and bacterial and phage dose can affect phage efficiency [[13], [14], [15]]. Since there is no centralized information about the effect of different process factors on phage function and efficacy, the aim of this study was to evaluate these factors on phage efficacy for reducing *E. coli* O157:H7 in different vegetables. Therefore, we conducted a systematic literature review to identify all related works on the topic, and then used a modeling approach to evaluate the parameters that have shown the most beneficial effect of phages on reducing *E. coli* O157:H7 in different vegetables from this selected literature.

## 2 Methods

### 2.1 Definition and literature search

Three global data bases including “PubMed”, “Scopus”, and “ScienceDirect” were employed to search the effects of phages on reducing *E. coli* O157:H7 in vegetables. The following key words were used to systematic search on subject, abstract and key words: *E. coli O157:H7*, phage, bacteriophage and vegetables. The related articles were respectively included based on titles, abstracts and full texts.

### 2.2 Inclusion/exclusion criteria

Among the extracted studies, the papers in non-English language, review papers, those studies without available abstracts or full texts, the articles reported as non-primary or treatments researches, the abstracts represented in the congresses, chapters of books, the irrelevant assays to effect of phage on E*. coli* O157:H7 reduction in vegetables and finally, those articles with no clear data were excluded from the list of assessed studies. The papers of reducing number of *E. coli* O157:H7 in vegetables, papers using the phage cocktails and experimental studies were also applied. However, articles focused on the influence of phages combined with other antimicrobial agents were omitted. The screening of eligible studies from data bases was performed.

### 2.3 Data analysis

All data were analyzed using Stata software (version 13.0). The reduction in the recorded *E. coli* O157:H7 was presented descriptively (mean ± SE). To predict the regression coefficient of *E. coli* O157:H7 reduction, univariate linear regression models were used. For eliminating potential confounders, the variables with significance level <0.20 were entered into the multivariate linear regression model. The variance inflation factor (VIF) was used in the multivariate regression model to remove the linearity effect. Therefore, variables with a VIF greater than four were eliminated. This equation (y_¡=β ^_0+β ^_1 X_¡+e_¡) was used to predict Salmonella reduction. R^2^ was used as a criterion to select the best model. *p* < 0.05 was considered as the significance level for the two-sided tests.

## 3 Results

### 3.1 Eligible studies and characteristics

**Fig. 1:**
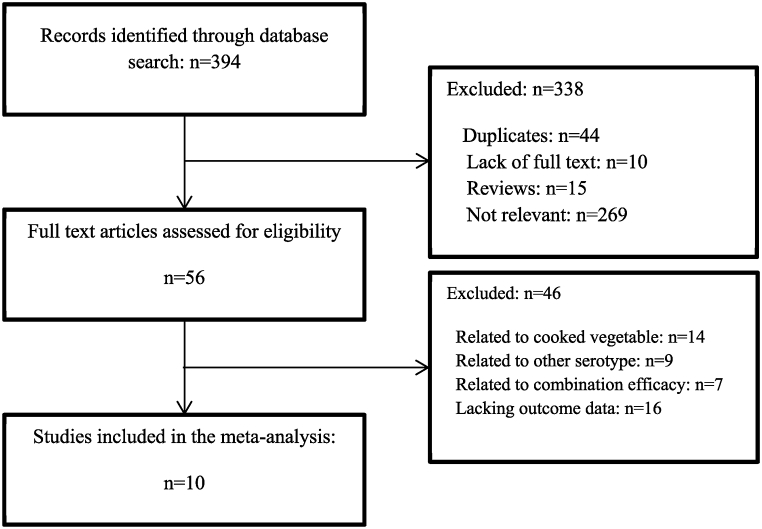
Flow chart of the systematic literature research.

The initial search identified a total of 394 potentially relevant studies, of which 56 studies were further evaluated. Finally, 10 articles were included in this study, after applying the exclusion criteria detailed before (Fig. 1). The baseline characteristics of the included studies are shown in Table 1. The main aim of this study was to investigate the level of *E. coli* O157:H7 reduction by utilizing bacteriophages in vegetables. All studies in this field were carried out between 2008 and 2021. Factors that were examined in this experiment as variables include: time, temperature, phage and bacterial dose, method of phage application, phage origin (commercial phage: commercially produced and supplied by companies and wild phage: isolated and identified by the researcher) and the type of vegetables being treated. The effect of phages on bacterial reduction was studied in the temperature range of 4–37 °C and in the time range of 0–168 h. The maximum dose of phage that was inoculated on vegetables in different ways was 9.8 log PFU/ml and the result indicated that the use of phage is effective in reducing targeted bacteria ranging between 0.1 and 6.9 log CFU/cm^2^.

**Table 1:** Characteristics of the included studies on *E. coli* reduction factors investigated in vegetables.

Reference	Objective	Factors investigated (dose in PFU/g; Temperature in °C; time in h)	Result
[8]	Determine whether treatment with *E. coli O157:H7*-specific phages significantly reduces the number of viable *E. coli O157:H7* cells	Phage dose: 10^9^ Temperature: 10 Time: 24, 120, 168	Naturally occurring bacteriophages may be useful for reducing contamination of various hard surfaces, fruits, vegetables, and ground beef by *E. coli O157:H7*
[16]	Investigate the decontamination efficiency of a lytic bacteriophage, M8AEC16, on nalidixic acid resistant and sensitive *E. coli O157:H7* strains in a ready-to-eat salad model	Temperature: 4, 10, 22 Time: 0.5, 1, 3, 5	The highest reductions were observed at 22 °C storage conditions. Reductions reaching up to 2.7 log cfu/g of viable *E. coli O157:H7* counts were observed in the beginning of the storage period. Phage M8AEC16 can be used as a biocontrol agent in the decontamination of *E. coli O157:H7* in such mayonnaise based ready-to-eat salads
[17]	The effectiveness of using bacteriophages (EcoShield™) to prevent contamination of lettuce with *E. coli O157:H7*	Phage dose: 9.8, 8.3 Temperature: 4 Time: 0, 24, 48, 72, 96, 120	Spray application of lytic bacteriophages to lettuce was more effective in immediately reducing *E. coli O157:H7* populations fresh cut lettuce
[1]	To investigate the ability of a bacteriophage cocktail to act as a natural intervention to lyse *E. coli O157:H7* on spinach leaves	Time: 0, 3, 6, 9, 12	The bacteriophages can be effectively used as a tool in reducing *E. coli O157:H7* contamination on fresh produce
[3]	To investigate the ability of a two-step biocontrol process utilizing lytic bacteriophages and a PW to inactivate *E. coli O157:H7*	Temperature: 25 Time: 24	The use of bacteriophage alone has been effective for reducing *E. coli O157:H7* in foods. *E. coli O157:H7* was able to grow back in broccoli after the initial kill during storage at 10 °C for 5–7 days. After 24 h, the phage cocktail had reduced the *E. coli O157:H7* population in broccoli by 99.5 %, and reduction was maintained at 99 and 97 % after 5 and 7 days, respectively.
[18]	The efficacy of bacteriophage BPECO 19 in eliminating or reducing biofilms on food and food contact surfaces	Temperature: 25, 20, 10	The phage treatment reduced cell viability by ≥ 2 log CFU/cm2 in biofilms grown on lettuce.
[19]	To examine the effectiveness of bacteriophages specific for *E. coli 0157:117* in reducing populations on fresh-cut lettuce	Temperature: 4 Time: 24, 48	Populations of *E. coli 0157:H7* on lettuce treated with ECP-100 on 0, 1 and 2 clays lower than those treated with the control
[20]	To isolate and select a phage that effectively inactivates pathogenic *E. coli* on different fresh produce.	Temperature: 25, 4 Time: 4, 24, 72, 48	The optimized phage treatment decreased the populations of pathogenic *E. coli* by 3.4–3.5 log CFU/g on spinach leaves.
[9]	To determine the effect of phage BEC8, on the viability of a mixture of *EHEC O157:H7* strains applied on leafy green vegetables	Temperature: 4, 8, 23, 37 Time: 0.16, 1, 24	*E. coli O157:H7*-specific phages against foodborne pathogens that may contribute to prevent future cases of bloody diarrhea
[12]	To confirm that the new phage preparation was also effective for managing the levels of STEC in high-risk foods	Phage dose: 10^6^, 5 × 10^6^, 10^7^	Spraying of phages onto foods experimentally contaminated with high pathogen levels resulted in only ca. 0.5- to 1-log reductions in contamination levels.

### 3.2 Univariate analysis

Table 2 presents the regression coefficients of the association for each exposure variable with the presence of *E. coli* O157:H7 reduction. The univariate regression coefficients were significant for most cases. The results of univariate analysis showed that for each unit of increase in temperature, time and method of phage application, the rate of reduction of *E. coli* O157:H7 bacteria increases by 3 %, 0.5 % and 50 %, respectively (p < 0.05). It was also found that bacterial and phage doses and origin of phages in the univariate model did not have a significant effect on the reduction of *E. coli* O157:H7 (p > 0.05). Regarding the role of different vegetables, our result showed that, the rate of bacterial reduction increased by spinach (58 %), cantaloupe (138 %), broccoli (124 %) and strawberries (345 %).

**Table 2:** Univariate and multivariate model for regression coefficient of *E. coli* reduction in vegetables.

Univariate model					Multivariate model			
variable	Beta	95 % Cl		P value	Beta	95 % Cl		P value
		Lower Upper				Lower Upper		
**Bacterial Dose**	0.07	−0.03	0.18	0.191	−0.10	−0.23	0.037	0.152
**Phage Dose**	−0.05	−0.15	0.04	0.290	0.06	−0.10	0.23	0.447
**Temperature**	0.03	0.01	0.04	0.000	0.03	0.02	0.043	0.000
**Time**	0.005	0.001	0.009	0.007	0.004	0.00	0.00	0.027
**Method of phage application**								
*Spray*	Ref	–	–	–	–	–	–	–
Immersion	0.50	0.27	0.73	0.000	0.94	0.60	1.29	0.000
**Origin of phages**								
Wild	Ref	–	–	–	–	–	–	–
Commercial	0.16	−0.12	0.45	0.270	−0.99	−1.55	−0.42	0.001
**Type of vegetable**								
Lettuce	Ref	–	–	–				
Spinach	0.58	0.25	0.90	0.001	0.50	0.21	0.80	0.001
Salad	0.01	−0.33	0.36	0.930	0.89	0.29	1.49	0.004
Cantaloupe	1.38	0.83	1.93	0.000	2.01	1.35	2.67	0.000
Broccoli	1.24	0.34	2.15	0.007	0.58	−0.28	1.46	0.186
Tomato	0.18	−0.85	1.22	0.720	−0.34	−1.39	0.70	0.521
Strawberry	3.45	1.68	5.23	0.000	2.38	0.82	3.95	0.003

### 3.3 Multivariate analysis

Table 2 presents the results of multivariate regression model. Since all of variables were highly correlated, called co-linear variables, the multivariable model was presented separately for each of these exposures. All variables except bacterial dose and phage dose were significantly effective on *E. coli* O157:H7 bacteria reduction (p˂0.05). The results of the multivariate analysis show that for each unit increase in temperature increases the rate of bacterial reduction by 3 %. These changes are statistically significant (Beta = 0.03; p = 0.000). Also, with the increase of each unit of time (h), the rate of *E. coli* O157:H7 bacteria reduction (0.4 %) increases significantly (p = 0.027). Regarding the method of phage application, the results showed that the spray application method reduces the number of bacteria by 94 % more than the immersion method (Beta = 0.94; p = 0.000). Regarding the origin of phages, the results showed that commercial phage is more effective on bacteria reduction by 99 %. Compared to lettuce, spinach and ready-to-eat salad, cantaloupe and strawberry significantly increased 50 %, 89 %, 201 % and 238 % reduction of *E. coli* O157:H7, respectively (p < 0.05), although in the case of vegetables, such as broccoli and tomatoes, no difference was found between them and lettuce for bacterial shedding (p > 0.05).

## 4 Discussion

This is the first time that a multivariate analysis and modeling approach has been applied for the investigation of the effect of various factors on phage reduction of *E. coli* O157:H7 regarding vegetables. The results showed that various factors such as temperature, time, method of phage application, origin of phages used and the vegetable type reduce the amount of *E. coli* O157:H7 in plants. However, neither bacterial nor phage dose play a role in *E. coli* O157:H7 reduction.

Temperature is a vital factor in bacteriophage survival. It plays an essential role in adhesion, penetration, proliferation and latency. At sub-optimal temperatures, less genetic material from the phage penetrates bacterial host cells. Therefore, fewer can participate in the reproduction stage. Higher temperatures can increase the latency stage [21], so temperature is deemed an important factor in phage performance. The results obtained in the present multivariate analysis showed that for each unit of temperature (between 4 and 37 °C) increase, the reduction of *E. coli* O157:H7 in vegetables increases by 3 %. The results of several other researchers confirm our results. For example, in 2017, Cufaoglu et al. stated that phage performs better at 25 °C, because bacteria can easily multiply at this temperature. It was also said that all phage groups at refrigeration temperature (4 °C), had the lowest bacterial reduction. This is because this temperature, reduces the metabolic rate such that neither *E. coli* O157:H7 nor infecting phage can replicate [16]. Furthermore, in another study that examined the effect of combined incubation (initially 20 °C and then 4 °C) compared to incubation at 4 °C to evaluate the performance of phage in pepper, the results showed that due to the higher possibility of bacterial growth at 20 °C, the activity of lytic phages is facilitated, and hence, phage has a better effect at high temperatures [20]. However, in some studies, different results of phage function at different temperatures have been mentioned. For example, in a study conducted by Sadehkuzaman et al., 2017, it was reported that BPECO 19 bacteriophage can effectively reduce *E. coli* O157:H7 even at 10, 20 and 25 °C in lettuce biofilm, indicating that this phage can act effectively in a wide range of temperatures [18]. In another study, the results showed that *E. coli* O157:H7 phages were more effective in killing pathogens in cantaloupe and fresh lettuce at refrigerator temperature (4 °C) than at 20 °C, which is probably due to other, unexamined factors. Among the superior survival of phages at this temperature is that there will be more phages to infect *E. coli* O157:H7 at this temperature [19].

Another factor in reducing the amount of *E. coli* O157:H7 is the duration of phage exposure to the bacterium. In the present study, different times (between 0.16 and 168 h) were used to evaluate the effect of phages. The results of multivariate modeling showed that for each unit of time increase, the rate of reduction of *E. coli* O157:H7 increases by only 0.4 %. This indicates that with the increasing duration of contact between the phage and the target bacterium, the rate of bacterial reduction does not increase significantly. Extensive studies have confirmed the results of our study [[22], [23], [24]]. In a study by Chandi et al., the effect of EcoShield phage on beef steaks infected with *E. coli* O157:H7 at incubation times between 5 and 1440 min was investigated, to determine the effect of longer contact times on phage efficiency. The results showed that the greatest effect of phage occurs during the first 5 min and the rate of reduction of *E. coli* O157:H7 was the same for all subsequent periods (94 %–98 %) [25]. Jassim et al. indicated that longer durations of exposure do not significantly increase the number of attacking phages [26]. Other studies have shown different results, so that in a study conducted by Amarillas et al., the effect of bacteriophage along with the oral coating of chitosan, as a tomato package, was investigated. In this study, incubation times between 0 and 160 h were used to evaluate the effect of contact time between phage and bacteria. The results showed that the longer the incubation time, the lower the bacterial number. The starting number of bacteria on day one was ∼7 log CFU/mL and dropped to ∼2 log CFU/mL after 140 h [5]. The natural lytic cycle of a bacteriophage lasts 20–40 min [27]. This study contradicts the results of Abuladze et al., who stated that although the whole lytic cycle takes approximately 40 min, most bacteria are affected by phage in the first minute [8]. In this regard, some researchers believe that the lysis of bacterial cells during the first hours of the experiment may be simply due to the high concentration of phage per target host cell which can lyse cells due to the destruction of the cell wall. This phenomenon is referred to as ‘lysis from without’. This lysis from without is the most likely pathway for biological control of foodborne pathogens, mainly at normal/cold storage temperatures [5].

According to our hypotheses for this study, the method of phage application to the sample surface was one of the most important factors for phage effecting *E. coli* O157:H7 reduction on vegetables. In the present study, both spraying and immersion methods were used to apply phage on bacteria located on vegetables, and the analysis confirmed that the spraying method was 94 % more effective than immersion. In a similar study, Ferguson, 2013, states that both immersion and spraying methods protect lettuce against *E. coli* O157:H7*,* but the use of lytic phages as a spray is faster in reducing the population of *E. coli* O157:H7 in lettuce [17]. Ferguson acknowledges that dipping lettuce in a phage solution may have impeded the mobility of the bacteriophage on the surface of the lettuce pieces, while spraying phage particles on the surface of food particles does not impede the mobility of the bacteriophage on the surface. In fact, this means that immersion may not be the optimal mechanism to distribute phages on the surface of lettuce. In other words, in the spraying method, phages are actively placed on the surface of lettuce and do not penetrate deep into the tissue of the product, so this facilitates the rapid interaction with *E. coli* O157:H7 cells [17]. Spraying ECP-100 phage on lettuce was able to rapidly reduce *E. coli* O157:H7 by 1.92 log CFU/cm^2^ compared to cantaloupe, which was pipetted. This is because spraying phage on the infected surface of lettuce can increase the contact of phage and *E. coli* O157:H7 bacteria [19]. Other researchers have reported that spraying specific phages of *Listeria monocytogenes* on freshly cut melons significantly reduced the bacterium compared to immersing contaminated melons in the phage mixture [28].

Contrary to our a priori assumptions, the dose of phage in the univariate model was negative. That is, for each unit increase in phage dose, the bacterial reduction rate decreases by 0.5 %. However, in the multivariate model, this value indicates a 6 % increase; nevertheless, the results of neither of these two models were significant (p > 0.05). Several studies have been performed by other researchers, the results of which confirm the results of our study. In other similar studies, researchers have concluded that the higher the number of phages, the better it can be at reducing pathogens [1,8,29,30]. Also, some studies, stated that the higher the initial concentration of phage at the time of application, the higher the bacterial reduction [[31], [32], [33], [34]], but in a study by Cofaoglu et al., 2017, it was found that the maximum bacterial reduction at the initial concentration phage occurs between 0.5 and 5.4 log and the use of higher concentrations of phage has no significant effect on bacterial reduction. They suggested that due to the costs of phage application, fewer of them could be used to kill food pathogens [16].

Sillankorva et al., 2012, state that phages can be isolated from different origins, such as food surfaces. In fact, they are present in a wide range of different habitats; for example, coliphages are a large group of phages in the feces [5,35,36]. In the present study, we divided the origin of phages into two categories: commercial and wild-type. From the analysis, we concluded that commercial phage category was more effective than the wild-type phage category. The reason for this is that commercial cocktails use several phages and also commercial phages are prepared with higher purity [37].

The results of multivariate model showed that the bacterial dose had no effect on the *E. coli* O157:H7 reduction by phage (p > 0.05). Cufaoglu et al., 2017, obtained similar results to our study. Their results showed that the rate of reduction of bacteria by phage at different initial concentrations of bacteria (3.9 and 5.9 log) is similar [16]. However, other studies have reported conflicting results, suggesting that phages will perform better at higher concentrations of initial bacteria [38].

Finally, in this study, we applied an alternative category for our modeling, which was based on the type of vegetables. The results of multivariate analysis showed that compared to lettuce, ready-to-eat salads and spinach have a greater ability to allow bacterial elimination by phages. In general, we concluded that green leafy vegetables have probably been more successful in this regard, due to having a wider surface for better phage function and having a higher contact surface with bacteria [39]. In the classification, the highest percentage of bacterial reduction was reported for strawberries and cantaloupe.

## 5 Conclusions

In summary, the result of this study indicate various factors such as temperature, time, method of phage application and type of vegetables can play an important role in the phage bactericidal efficacy. To better understand this issue, more research and studies in this area are necessary, since our work included only 10 eligible manuscripts. The statistical significance of some factors may be seriously influenced by the lack of studies on their effect; hence we suggest exercising caution before generalization of the findings of the present work. In addition, this subject has to include other process conditions, such as combined treatment method, as well as the effect on various other vegetables.

## Ethics approval and consent to participate

Not applicable.

## Availability of data and materials

The datasets used and/or analyzed during the current study available from the corresponding author on reasonable request.

## Funding

This study was entirely financed by 10.13039/501100006096Shiraz University of Medical Sciences (SUMS), Shiraz, Iran with project number 24822 and ethical cod IR. SUMS.REC.1400.835.

## CRediT authorship contribution statement

**Maryam Safarirad:** Writing – review & editing, Writing – original draft, Software, Methodology, Investigation, Formal analysis, Data curation, Conceptualization. **Mohsen Shahdadi:** Writing – review & editing, Writing – original draft, Validation, Investigation, Formal analysis, Data curation, Conceptualization. **Enayat Berizi:** Writing – review & editing, Writing – original draft, Supervision, Resources, Project administration, Investigation, Funding acquisition, Conceptualization. **Seyed Mohammad Mazloomi:** Writing – review & editing, Writing – original draft, Supervision, Project administration. **Saeid Hosseinzadeh:** Writing – review & editing, Writing – original draft, Supervision, Data curation, Conceptualization. **Maryam Montaseri:** Writing – review & editing, Writing – original draft, Software, Formal analysis, Conceptualization. **Zahra Derakhshan:** Writing – review & editing, Writing – original draft, Validation, Supervision.

## Declaration of competing interest

The authors declare that they have no known competing financial interests or personal relationships that could have appeared to influence the work reported in this paper.
